# The socio-economic impacts of Covid-19 restrictions: Data from the coastal city of Mombasa, Kenya

**DOI:** 10.1016/j.dib.2020.106317

**Published:** 2020-09-18

**Authors:** Justus Kithiia, Innocent Wanyonyi, Joseph Maina, Titus Jefwa, Majambo Gamoyo

**Affiliations:** a Coastal and Marine Resources Development (COMRED), Kenya; b School for Field Studies- MA, United States; c Macquarie University, Australia; d Technical University of Mombasa, Kenya

**Keywords:** Covid-19, Mombasa, Cities, Gender, Socio-economic impacts, Restrictions, Curfew

## Abstract

The novel corona virus disease (Covid-19) outbreak has caused great uncertainty in all spheres of human life. The experience has been incredibly humbling given that no country or section of society, regardless of its wealth or status, has been spared. The pandemic is not only a health crisis, but is also having serious damaging effects on societies, economies and vulnerable groups. Timely response is necessary in order to alleviate human suffering and to prevent irreversible destruction of livelihoods. This paper provides preliminary data on the socio-economic impacts of Covid-19 in the coastal city of Mombasa, Kenya, at the time of government-imposed curfews and cessation of movement. We conducted online surveys for two weeks during the restrictions period. The data was collected using online questionnaires directed at the city residents. The data highlights the mobile gender gap resulting from gender inequalities, residents’ reliance on the government for Covid-19 information but lack of trust for government interventions, inadequate provisions of essential services, and the residents’ lack of preparedness to tackle similar challenges in the future.

## Specifications Table

**Table:** 

Subject	Social Sciences
Specific subject area	Health, Social Sciences (General)
Type of data	Table Chart Graph Figure
How data were acquired	A survey questionnaire was developed to elicit information on various socio-economic aspects of the Covid-19 pandemic. The questionnaire consisted of 22 closed and open-ended questions to leverage the balance between obtaining quick responses to straight forward questions and probing for depth. A total number of 103 respondents completed the survey.
Data format	Raw Analysed
Parameters for data collection	The survey was distributed to residents across all 5 sub-counties of Mombasa, namely, Mvita, Nyali, Kisauni, Likoni and Changamwe (figure 1). Survey questionnaires were sent to residents who were 18 years.
Description of data collection	The online survey was distributed to contacts and contacts of contacts in a manner that is consistent with the snowball and convenient sampling techniques. Contacts were asked to share the survey widely with their networks. The survey was conducted from 20th May to 1st June 2020.
Data source location	Coastal and Marine Resources Development (COMRED) Nyali, Mombasa, Kenya The data was collected from the coastal city of Mombasa, situated at 4°0’S, 39°36’E and 18 ft above sea level. Mombasa.
Data accessibility	Raw data is available at COMRED's repository and has also been shared with Data in Brief journal.

## Value of the Data

• The data provides a snapshot of the socio-economic impacts of Covid-19 on Mombasa city residents. Analysed along key demographic aspects such gender shows disparities in the impacts of Covid-19, among residents, in terms of access to information, income differentials and livelihood options.
• This data is vital in helping authorities, partners and other stakeholders to tailor responses to facilitate recovery from the crisis. For instance, it can support local and national authorities in addressing the challenge of dissemination of mis and disinformation about Covid-19, by formulating specific information on policy responses and specific behaviour to be adopted.
• As the pandemic is still evolving, this data can be used as a baseline for continuous data gathering, analysis and interpretation to provide new insights about the disease. The survey can be further developed to gather data on the residents’ access to essential services, such as running water, during Covid-19 or similar pandemics.

## 1 Data Description

The gathered information is general in nature and was meant to provide a preliminary assessment of the socio-economic impact of Covid-19 during restrictions. However, we isolated gender issues for further analysis. This was dictated by the data, whose casual examination revealed gender disparities. Consequently, we divided our results into two categories, namely, i) gendered social-economic impacts and ii) general socio-economic impacts. Our data did not return significant values across all variables when analyzed against age.

### 1.1 Gendered socio-economic impacts

Throughout history, pandemics have been known to have differential impacts on women and men. Because of this, the draft resolution from the 146th session of the WHO executive Board recommended that strategies for Covid-19 preparedness and response must be grounded in strong gender analysis [12]. The WHO has been calling for gender-sensitive research on adverse health, social and economic impacts of Covid-19. Hence, disaggregating data by sex and age would help in understanding the social and economic impacts of Covid-19 on women and men.

According to the 2019 Kenya Population and Housing Census report, the gender composition in Mombasa is nearly 50-50 (50.5% male and 49.5% female) [3]. Participants in this survey comprised of 40.8% female and 59.2% men with no other gender identified. While the gender differentiated response might have been occasioned by our sampling technique, it is most likely an indication of the underlying gender inequality. For example, survey respondents required internet to access to complete and return the survey. However, while men have long been able to surf the web, many women in Africa lack access to the internet, even though they might have smartphones. Many women lack enough money to use the internet, with 7 out of 10 online mobile users being men. In Kenya, only 26% of women use mobile internet [2]. Employment is in many cases a determinant of household disposal income which in turn determines whether individuals can afford to pay for internet or not. Yet in Mombasa, 26% of women are not economically engaged, and only 15% are employed while men make up 15% and 29% of the two categories respectively [5].

A survey report on the ‘socio-economic Impact of Covid-19 on Households’ released by the Kenya National Treasury Office, in May 2020, revealed that more women than men work in jobs that are vulnerable to disruption, especially in the service and manufacturing industries [4]. Our survey showed that only about 11% of the women in self-employment could work from home and of the 28% of the women in casual employment, none could earn a living by working from home (Table 1). This points to the type of self-employment that women are involved in. A previous report had shown that women tended to operate smaller businesses associated with traditional women's roles such as hairdressing, selling fish, trading in vegetables, operating small kiosks and so on, in order to provide for their families (see [8]). Hence, these require face-to-face interactions.

**Table 1:** Employment type and ability to work from home during the Covid-19 curfew and cessation of movement in Mombasa.

Employment Type	% sampled population	% female	% sampled pop working from Home	% female working from home
Full-time employment	59.2	44.3	54.1	55.6
Self-employment	26.2	33.3	25.9	11.1
Casual employment	6.8	28.6	0	0
Other employment	7.8	N/A	N/A	N/A

Those who worked from home expressed various challenges.Fig. 2 shows some of the challenges mentioned by this group. The challenges related to access, reliability and affordability of internet services. Overall, problems associated with internet for those who worked from home accounted for 31%. This points to the low level of connectivity and affordability of internet services in Mombasa. Another challenge was distractions, which was mentioned by 29% of the respondents. Many respondents attributed the distractions to multi-tasking as they home-schooled their children while completing work related tasks. Others found the home environment unconducive for work due to limited spaces. The problem of space was further confirmed by our data, which shows the average household size to be 4.5 people. This is much higher than 3.1 people per household reported for Mombasa by the Kenya National Bureau of Standards, population census report [3]. However, it is worth noting that KNBS obtained its figure by dividing the city's total population with the number of households. Our figure of 4.5 people per household is consistent with the findings that African women have 4.5 children on average. It could also be explained by the fact that Mombasa has a high population density of 5495 persons per square kilometre [3,10]. The high average household size coupled with the small house sizes explains why the respondents found the home environment to be unconducive for work.

**Fig. 1:**
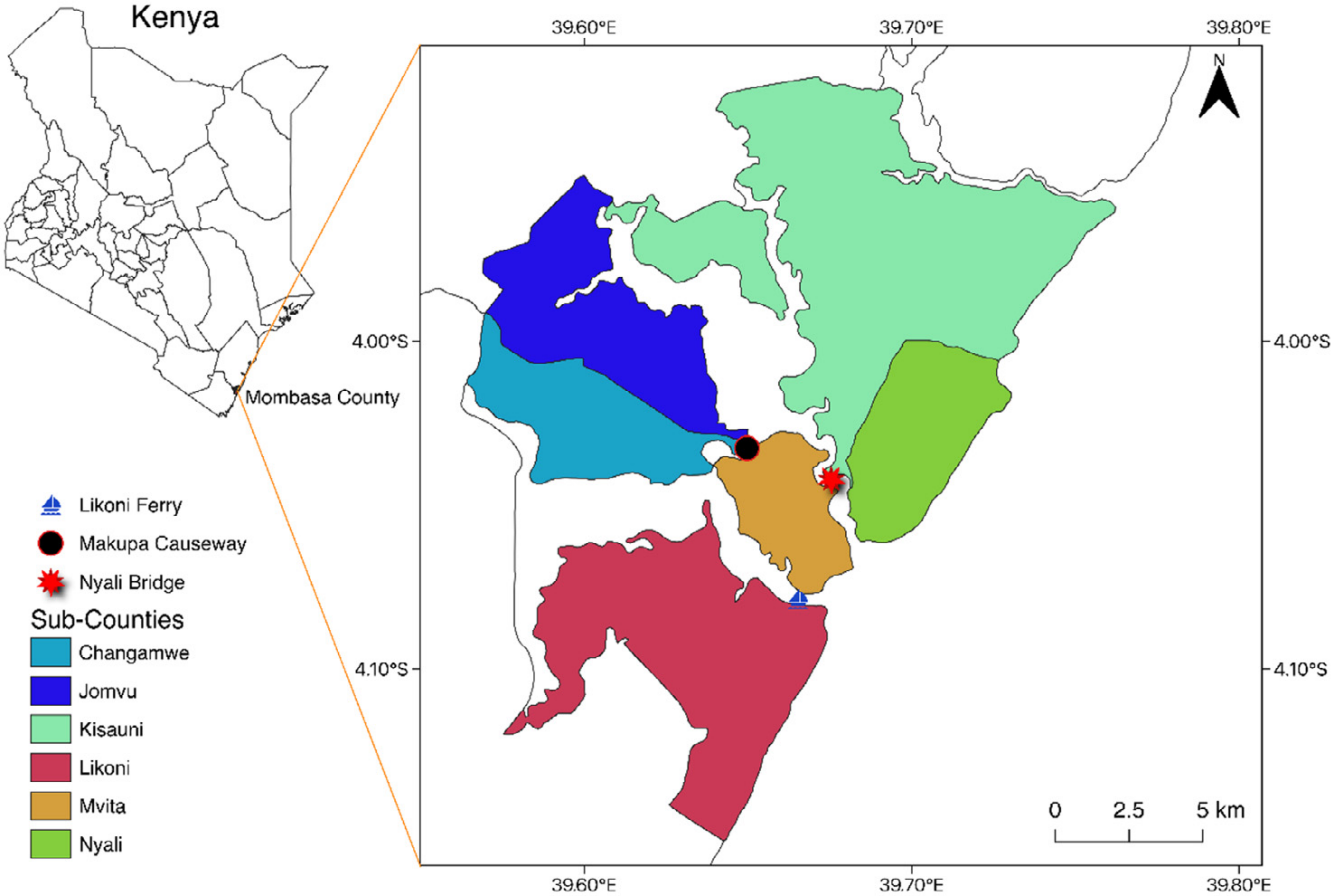
Map of Mombasa County showing the sub-counties and transport infrastructure connecting the island (Mvita) with the mainland.

**Fig. 2:**
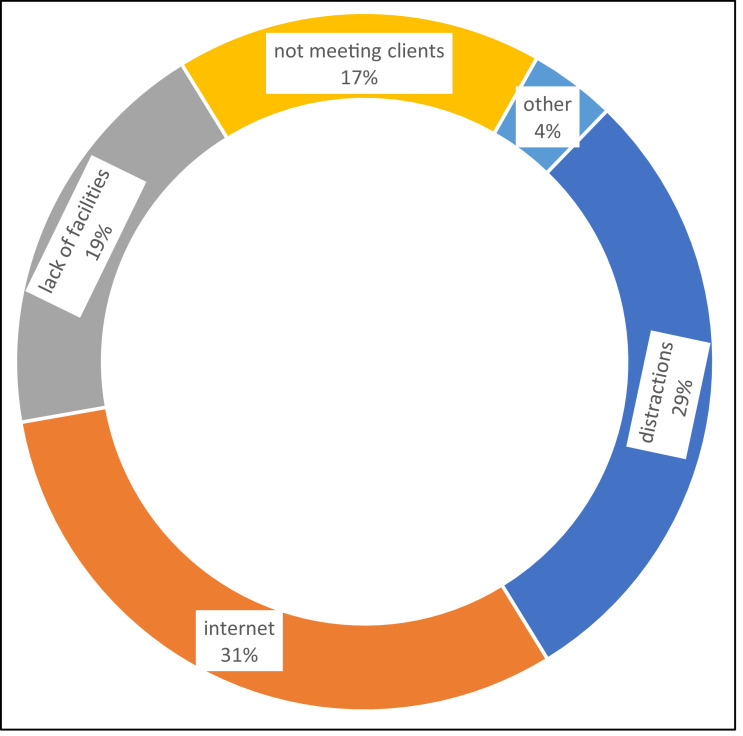
Challenges faced by residents who worked from home.

The women's lack of access to internet is an additional stratifier of the gender inequalities that could exacerbate adverse differential impacts of Covid-19. For example, this might mean that women are missing out on credible Covid-19 information that is shared through social media networks and other internet sources. Women comprised of about 35% of those who use social media and online research for Covid-19 information, which is the close to Fröhlich [2] finding that 7/10 online mobile users in Africa are men.

**Fig. 3:**
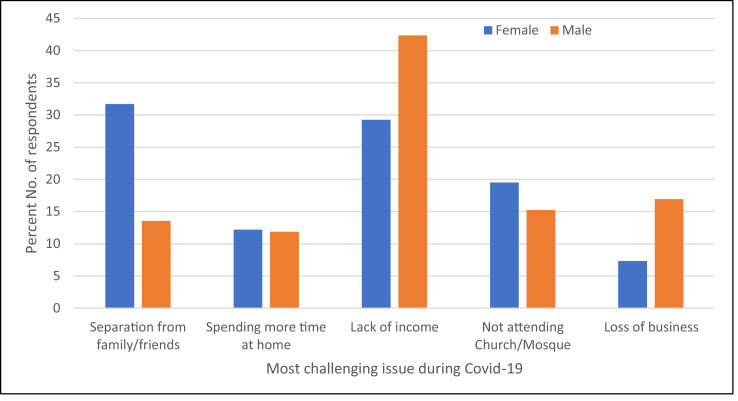
Most challenging issues during Covid-19 lockdown.

Gender differential was also evident regarding the most challenging issues during restrictions. Male respondents were mostly experiencing lack of income and loss of business at 42% and 15% respectively. On the other hand, most female respondents reported facing challenges of separation from family/friends and not attending religious gatherings (Fig. 3); pointing to gendered power relations which depict women's relational interests and men's preoccupation with the pursuit of personal growth.

### 1.2 General socio-economic impacts

In addition to identifying the challenges presented in Fig. 3, majority of the respondents said that the restriction of their movement had grossly interfered with their day to day activities. Not surprising, 31% of the respondents specifically identified financial loss as a major consequence of the Covid-19 measures. Some respondents (7%) felt their expenses were higher as a result of the restrictions. Though not in significant numbers, it is noteworthy that some residents reported feeling stressed and having to incur additional expenses due to price hikes of some essential commodities, and the continuous presence of children at home owing to school closures (see Table 2).

**Table 2:** The impact of Covid_19 restrictions on individuals and/or households.

Effects of Covid-19 restrictions	Percent
Financial loss	31
Restricted movement	39
Stress	6
Restricted religious activities	4
Loss of education for children	6
Expensive	7
Others	7

**Fig. 4:**
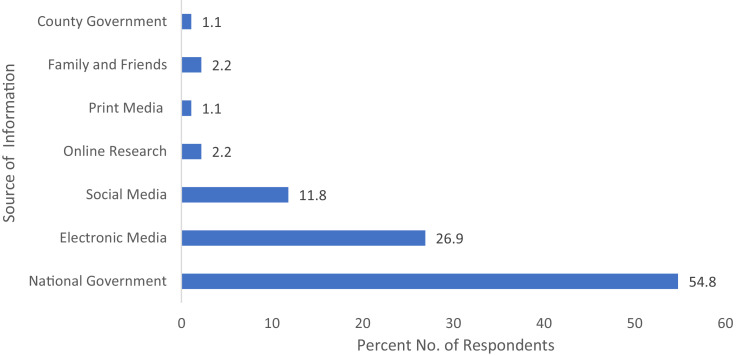
Residents’ sources of covid-19 information.

Similar to other crisis situations, the Covid-19 pandemic has raised important issues around information dissemination. Our survey showed that approximately 55% of the residents relied on the national government for Covid-19 information (Fig. 4). This was expected given the national government has been providing press briefings every day since the first case was reported. These briefings are broadcast live on national TV and Radio stations. Electronic media was the second most common source of information at 26.9%. This means that some of the respondents who identified electronic media as their source of Covid-19 got their information from the government. Electronic medial included TV and Radio; Social Media included Facebook, WhatsApp, Twitter, and Instagram (Fig. 4).

When asked whether they found Covid-19 messages conveyed by the government easy or difficult to follow, 20% of those who said the messages were difficult to follow gave the reason that it was difficult to practice social distancing in crowd places. Another 24% mentioned the government's mixed messaging, which led to confusion and conflicting information. The government has done little to address infodemics. One of the myths which went viral within Mombasa was that of the mystical tea. The coastal region was rife with rumours that black tea without sugar, drunk before dawn, could both cure and prevent coronavirus. This information was attributed to a child who was born at night, and uttered words about black tea before dying [9]. The spread of such rumours is not unexpected given the usual tendency for fringe ideas to acquire prominence when people become anxious and isolated without much social support.

**Fig. 5:**
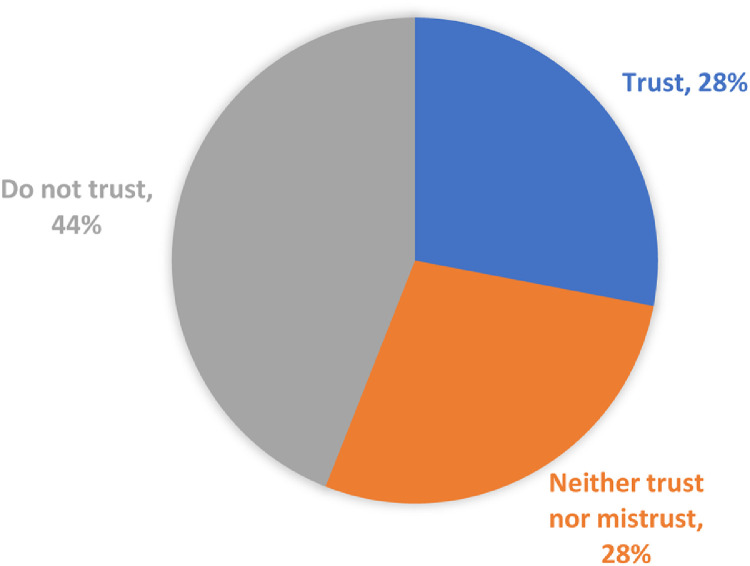
Trust for government to take care of vulnerable people.

It was interesting to note that while a higher percentage of Mombasa residents relied on the national government for Covid-19 related information, there was a significantly low trust for the government to do what it was promising to do with regard to taking care of the vulnerable people. 44% of the respondents stated that they did not trust the government (Fig. 5). This is despite the National government having announced an economic stimulus of KES 53.7 Billion to help keep the country afloat, and effecting cash transfers under the National Safety Net Programme (NSNP) as well as local relief food distribution efforts by the Mombasa County government.

**Fig. 6:**
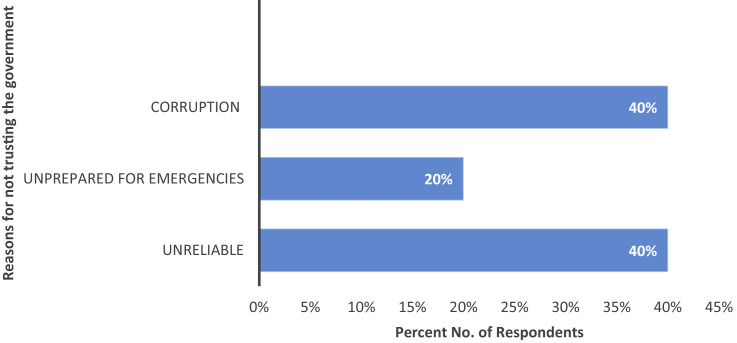
Reasons for not trusting the government to care for the vulnerable people during Covid-19.

Those who did not trust the government to care for the vulnerable gave various reasons for not doing so. The reasons fitted into one of the following 3 categories, namely; government is corrupt, government is unreliable and government is unprepared to deal with emergencies of the scale of Covid-19 (Fig. 6). Many Africans assess their public officials skeptically, wary of corruption, coercion and inadequate care for ordinary people's physical, economic and social welfare [1].

Mombasa is a world-famous tourist city, as exemplified by the number of Hotel-Bed nights. The combined Hotel-Bed nights in the region stood at 3904 in 2019. This accounted for about 43% of the total occupied beds in the entire country [5]. While Covid-19 has affected all business in all sectors, the tourism industry has been the hardest hit, especially due to the local restriction of movement and cessation of international travel. According to the KNBS survey, accommodation and food services had a high variance of 30 hours, compared to the usual hours of work per week [4]. In April 2020, the Kenya Tourism Federation (KTF) reported some hotels on the coast having occupancy rates below 10%, necessitating close down and sending staff on unpaid compulsory leave [11].

In terms of service provision during Covid-19 restrictions, access to running water was imperative because the number one recommended protective measure against the virus is frequent washing using soap. Our data does not determine whether the respondents referred to the availability of running water, standpipes or water kiosks. However, water statistics in Mombasa show that the demand for portable water per day is 200,000 m^3^. The piped system is only able to supply a maximum of 50,000 m^3^. The rest of the water comes from boreholes and other unidentified sources [6].

**Fig. 7:**
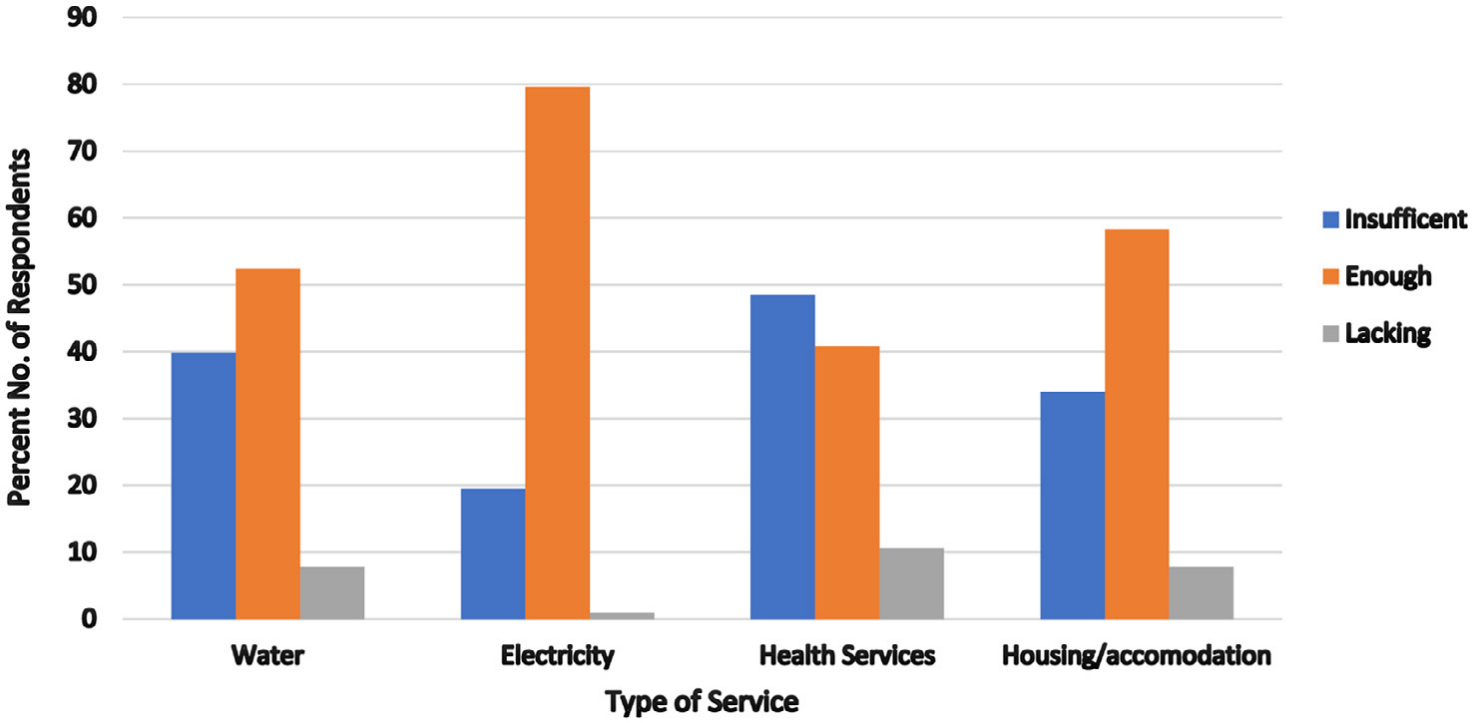
Residents perception on the provision of essential services.

The provision of electricity, health care and accommodation/housing services is also vital. For example, with the increasing number of cases, the demand on health facilities and health care workers would increase leaving health systems overstretched and unable to operate effectively. According to WHO, previous outbreaks have demonstrated that when health systems are overwhelmed, mortality from vaccine-preventable and other treatable conditions can also increase dramatically [12]. The surveyed residents adjudged health services to be insufficient, and in some cases, completely lacking (see Fig. 7). It is unclear how our data might have been affected by the respondents’ misunderstanding of the functioning of the health care systems during this time of Covid-19. However, it must be remembered that over the years, Kenya has relied on external funding for the implementation of health interventions, with government allocation to health still at 6%, way below the international benchmark of 15% [7].

The government's ‘stay home orders’ brought to the fore the importance of adequate housing/accommodation provision in the city. Yet, Mombasa has the second-highest number of street individuals (1809), only second to Nairobi with 6743 (KNBS, 2020). The respondents concern about inadequate housing are captured in our data, whereby nearly 35% of the respondents have described housing/accommodation as ‘insufficient’ (Fig. 7). This also agrees with our findings on large household sizes (4.5 people) meaning that even where residents have accommodation, this may not be adequate to cater for all the individuals in the household.

The question of whether or not people have learnt from crisis is often posed after every crisis event. Lessons learned from crisis, if implemented, could lead to a better society. After the crisis, people tend to want to pick up, as quickly as, possible from where they left off, with little motivation to incorporate lessons learnt into structural processes for long term implementation. While the effects of Covid-19 are still unfolding, it would be interesting to know what lessons Mombasa residents will eventually learn from it, and how these would be incorporated into everyday processes. So far, our preliminary assessment shows that only 17% of the survey respondents felt that their Covid-19 experiences have prepared them to face similar crisis in the future. 57% were either unprepared or unsure of the extent to which their current experience has prepared them for future pandemics. However, this reflects our earlier findings that 59.6% of respondents were on full time employment, the rest likely being in informal sector or casual labour and therefore would do little to disrupt their normal life even after this pandemic.

## 2 Experimental Design, Materials and Methods

A survey questionnaire was developed to elicit information, from residents of Mombasa city, on various socio-economic aspects of the Covid-19 pandemic. The questionnaire consisted of 22 closed and open-ended questions to leverage the balance between obtaining quick responses to straight forward questions and probing for depth. The online survey was distributed to contacts and contacts of contacts in a manner that is consistent with snowball and convenient sampling techniques. Contacts were asked to share the survey widely with their networks. The survey was conducted from 20th May to 1st June 2020. Data was analysed using Microsoft Excel. It did not return significant values across all variables when analysed against age.

## Ethics Statement

All ethical issues which were likely to arise from the conduct of the study were considered as part of the preparation and administration of the survey. The survey was part of a broader study funded by WIOMSA and approved by the National Commission for Science, Technology and Innovation (NACOSTI) in Kenya. Survey questionnaires were only shared with those above 18 years who were willing to volunteer information.

## Declaration of Competing Interest

The authors declare that they have no known competing financial interests or personal relationships which have, or could be perceived to have, influenced the work reported in this article.

## Appendix Supplementary materials

Image, application 1

Image, application 2
